# Identification of a *Schistosoma japonicum* MicroRNA That Suppresses Hepatoma Cell Growth and Migration by Targeting Host *FZD4* Gene

**DOI:** 10.3389/fcimb.2022.786543

**Published:** 2022-01-31

**Authors:** Pengyue Jiang, Jing Wang, Shanli Zhu, Chao Hu, Yu Lin, Weiqing Pan

**Affiliations:** ^1^ Institute for Infectious Diseases and Vaccine Development, Tongji University School of Medicine, Shanghai, China; ^2^ Department of Tropical Diseases, Naval Medical University, Shanghai, China

**Keywords:** schistosomiasis, microRNA, hepatoma cell, cross-species regulation, *FZD4*

## Abstract

Previous studies have demonstrated miRNAs derived from plants and parasites can modulate mammalian gene expression and cell phenotype in a cross-kingdom manner, leading to occurrence of diseases or strengthening resistance of host to diseases such as cancer. In this study, we identified a schistosome miRNA (named Sja-miR-71a) through screening of 57 *Schistosoma japonicum* miRNAs that exerts antitumor activity *in vitro* and *in vivo* models. We demonstrated presence of this parasite miRNA in liver cells during infection. We showed that Sja-miR-71a arrested cell cycle at G0/G1 phase of hepatoma cell lines and inhibited cell proliferation *in vitro*. The HepG2 transfected with Sja-miR-71a mimics displayed significant reduction of migration and colony formation. Further, growth of the tumor cells transfected with the Sja-miR-71a mimics was obviously suppressed in a xenograft mouse model. Mechanically, we found the antitumor activity of Sja-miR-71a was through targeting a host gene encoding Frizzled Class Receptor 4 (FZD4), as *FZD4* small interfering RNAs (siRNAs) generated a similar inhibitory effect on the tumor. These data indicated that Sja-miR-71a is a tumor suppressor miRNA and suggested this parasite-derived miRNA as a potential therapeutic target for cancer.

## Introduction

The genus *Schistosoma* is the causative agent of schistosomiasis. The adult female parasite of *Schistosoma japonicum* (*S. japonicum*) in the mesenteric veins of hosts lays numerous eggs which are subsequently trapped in the liver and intestine. In previous studies, we first reported five conserved microRNAs within the parasite adults (Xue et al., 2008) and later we and the others applied deep sequencing to have identified large number of miRNAs, namely, those specific to *Schistosoma* and conserved miRNAs within the eggs and the other parasite stages (Huang et al., 2009; Wang et al., 2010). Further, schistosome miRNAs were also identified from the extracellular vesicles (EVs) released by both adult worms and eggs of *S. japonicum* (Wang et al., 2015; Zhu et al., 2016). Furthermore, studies on *Litomosoides sigmodontis*, *Heligmosomoides polygyrus* (Coakley et al., 2017; Cai et al., 2019), *Ascaris suum* (Hansen et al., 2019), *Schistosoma mansoni* and *Fasciola hepatica* (Ovchinnikov et al., 2020), also suggested that RNAs derived from the secretion products of diverse helminths are expected to be absorbed by host cells through transport in extracellular vesicles.

MicroRNAs (miRNAs) are a large group of naturally occurring small noncoding RNAs that regulate post-transcriptional expression of genes *via* binding to the 3ʹ-untranslated regions (3ʹ-UTR) of target mRNAs, which leads to either mRNA degradation or translational pausing (Gramantieri et al., 2008). Accumulated data indicated miRNAs play important roles not only in biological process such as cell proliferation, development or differentiation, but also in progression of many human diseases (Masaki, 2009; Ang et al., 2011). Numerous studies have demonstrated that miRNAs play crucial roles in cancer acting as oncogenes to promote carcinoma development or as anti-oncogenes to suppress tumor growth. For example, microRNA-155-5p promotes hepatocellular carcinoma (HCC) progression (Fu et al., 2017), while miRNA-7 inhibits HCC growth and metastasis (Fang et al., 2012). Therefore, targeting the miRNAs that regulate tumor-relevant genes could be potential therapeutics for cancer.

Recent studies revealed that exogenous miRNAs derived from plants or parasites can regulate target genes expression of recipient cells in a cross-species manner. A plant-derived miR-159 inhibited breast cancer growth by downregulation of *TCF7* expression and affected Wnt pathway (Chin et al., 2016). The schistosome-derived Sja-miR-2162 was delivered to hepatic stellate cells of mice infected with *S. japonicum* and promoted hepatic fibrosis through cross-species regulation of target gene, the host *Tgfbr3* gene (He et al., 2020). While several other schistosome-derived miRNAs, such as Sja-miR-3096 and Sja-miR-7-5p, exert antitumor activity through modulation of tumor-related genes. For example, the Sja-miR-3096 inhibited cell proliferation and migration of human hepatoma cells by targeting phosphoinositide 3-kinase class II alpha (*PIK3C2A*) (Lin et al., 2019). Therefore, discovery of such exogenous miRNAs, such as schistosome microRNAs, should be particularly interesting for development of novel tumor intervention. In this study, we further screened and identified the *S. japonicum*-derived miRNAs that have antitumor activity. We found that Sja-miR-71a, an *S. japonicum* miRNA, can arrest hepatoma cell cycle at G0/G1 phase and inhibited migration of tumor cells by targeting the *FZD4* gene.

## Materials and Methods

### Cell Lines and Culture

The HepG2, SMMC-7721 and hepa1-6 cell lines were purchased from the Shanghai Institute of Biochemistry and Cell Biology of the Chinese Academy of Sciences (Shanghai, China). The HepG2 and hepa1-6 was cultured in Dulbecco’s Modified Eagle’s Medium (DMEM; Gibco, USA) supplemented with 10% fetal bovine serum (FBS; Gibco) and 1% Penicillin–Streptomycin (Gibco), while SMMC-7721 was cultured in RPMI-1640 supplemented with 10% FBS and 1% Penicillin–Streptomycin. All the cell lines were cultured in a humidified atmosphere containing 5% CO_2_ at 37°C.

### Synthesis of miRNA Mimics and siRNA

The total of 57 miRNAs sequences were obtained from the reference (Huang et al., 2009). The *FZD4* siRNA sequences designed by the RNAi Designer website (Thermo Fisher) and negative control (NC) mimic were chemically synthesized and purified by Genepharma (Shanghai, China). The sequence of the miRNAs is provided in **Table S1**. All these unmodified mimics and siRNAs were purified by High Performance Liquid Chromatography (HPLC).

### Cell Transfection

HepG2, SMMC-7721 or hepa1-6 was seeded into 24-well plates, ensured 70% confluence at the time of transfection. Transfection was performed using Lipofectamine 3000 (Invitrogen) method following the manufacturer’s protocol. MiRNA mimics and negative controls were used at a final concentration of 40 nM, while siRNA were at the final concentration of 120 nM. At 48 h post-transfection, cells were collected for detection.

### RNA Isolation and Quantitative RT-PCR

Total RNA was extracted from the cultured cells or tissues using TRIzol Reagent (Invitrogen, USA) according to the manufacturer’s instructions. Assays to quantify Sja-miRNA-71a or mRNA were performed using ChamQ™Universal SYBR^®^ qPCR master mix (Vazyme, Nanjing, China) on 7500 real-time PCR system (Applied Biosystems). Briefly, 500 ng of total RNA was reverse-transcribed to cDNA using M-MLV reverse transcriptase (TaKaRa, Dalian, China) and a stem-loop RT primer. Since U6 small nuclear and GAPDH have been used as the internal control for miRNA and mRNA (Meng et al., 2007), miRNA and mRNA expression were normalized to their corresponding internal control genes and the relative change was calculated using the 2^−Δ^Ct and 2^−ΔΔ^Ct method (Livak and Schmittgen, 2001), respectively. All PCR experiments were done in triplicate. All the primers are listed in **Table S2**.

### Cell Cycle Measurement

HepG2, SMMC-7721 or hepa1-6 cells were transfected with miRNA or NC mimics respectively, three replicates per group. Cells were collected after 48 h and stained with propidium iodide (PI, Beyotime, China) for cell cycle analysis with FACS (BD, USA). Data were collected and analyzed with the FlowJo software.

### Cell Proliferation Assay

Cells were transfected with miRNAs, siRNAs or NC mimics respectively with four replicates per group. At 24 h post-transfection, cells (1.5 × 10^3^ cells) were collected and plated into 96-well plate for 24, 48, 72, and 96 h. At each indicated time, 10 µl Cell Counting Kit-8 reagents (Dojindo, Kumamoto, Japan) was added to each well and cells were incubated for 1 h at 37°C, then using the Microplate reader (Bio-Tek, USA) to measure the absorbance at 450 nm.

### Transwell Migration Assay

The migration ability of HepG2 and SMMC-7721 cells transfected with Sja-miR-71a or *FZD4* siRNAs and hepa1-6 cells treated with Sja-miR-71a was tested in a Corning Transwell Chamber (8-µm pore filter, 24-well cell cluster). The cells were harvested 24 h after transfection, suspended in FBS-free DMEM culture medium and added 100 µl to the upper chamber (1 × 10^4^ cells/well). At the same time, 700 µl of DMEM with 10% FBS was added to the lower compartment, and the Transwell containing plates were incubated for 24 h in a 5% CO_2_ atmosphere that was saturated with H_2_O. After incubation, cells that had entered the lower surface of the filter membrane were fixed with 4% paraformaldehyde for 25 min at room temperature, washed 3 times with distilled water and stained with 0.1% crystal violet in 0.1 M borate and 2% ethanol for 15 min at room temperature. Cells remaining on the upper surface of the filter membrane (non-migrant) were scraped off gently with a cotton swab. The lower surfaces (with migrant cells) were imaged using a photomicroscope (5 fields per chamber), and the cells were counted blindly. Three replicates per group.

### Colony Formation Assays

For the colony formation assay, cells were digested and counted after transfected with miRNA, siRNA or NC mimics at 24 h post-transfection, and then 200 cells were placed in each well of a 24-well plate and incubated at 37°C for 2 weeks, with three replicates per group. Clones were fixed and stained in a dye solution containing 0.1% crystal violet and 20% methanol, and the number of clones was counted.

### Plasmid Construction and Luciferase Reporter Assay

The sequences of Sja-miR-71a targets in human genes were predicted by software miRDB (Wong and Wang, 2015) and RNAhybrid (Kruger and Rehmsmeier, 2006). The predicted target genes were listed in **Table S3**. A dual luciferase reporter assay was carried out to determine if *FZD4* was the target gene of Sja-miR-71a. Briefly, a 500 bp sequence of the 3ʹ-UTR of *FZD4* gene, which contains either wild-type or its mutant at the binding site to Sja-miR-71a sequence were amplified by PCR or over-lap PCR with KOD plus neo (TOYOBO Life Science, Japan), and inserted into a pmirGLO luciferase reporter vector through *SacI* and *XbaI* restriction sites (Promega, USA). The primers are listed in **Table S2**. These two plasmids were designated as FZD4-WT or FZD4-MT, respectively. For the luciferase reporter assay, both HepG2 and SMMC-7721 cells were seeded into 48-well plates (3 × 10^3^ cells/well) and cultured in complete DMEM supplemented with 10% FBS at 37 ˚C and co-transfected with Sja-miR-71a mimics or NC mimics, the plasmid by Lipofectamine 3000, respectively. At 24 h post-transfection, cells were collected, Renilla and firefly luciferase activities were measured using a Dual Luciferase Assay system (Promega Corporation, USA). The activity was normalized to that of Renilla luciferase.

### Protein Extraction and Western Blotting

At 48 h post-transfection, the cells were washed with PBS (pH 7.4) and lysed using RIPA Lysis buffer (Beyotime, China) supplemented with a Protease and Phosphatase Inhibitor Cocktail (Thermo Scientific 78440) on ice for 30 min. The supernatants of cell lysates were collected, and measured for the protein concentration using a BCA protein assay kit (Beyotime, China). Western blotting was performed with the corresponding antibodies to analyze the protein. The antibodies were purchased from the following sources: anti-FZD4 (1:1,000 dilution, A02191-1, BOSTER, China); anti-β-ACTIN (1:1,000 dilution, Beyotime, China).

### *S. japonicum* Infection and Isolation of Hepatocytes

Animal experiments were performed in accordance with the Guide for the Care and Use of Laboratory Animals of the National Institutes of Health, and approved by the Internal Review Board of Tongji University School of Medicine (permit number: TJLAC-015-028). Cercariae of *S. japonicum* were provided by the National Institute of Parasitic Disease, Chinese Center for Disease Control and Prevention (CDC). All the procedures were described as previously (He et al., 2016; He et al., 2018).

### Tumor Xenograft Animal Model

Male athymic nude mice (n = 6) were housed and manipulated according to the protocols approved by the Shanghai Medical Experimental Animal Care Commission. The hepatoma cells were transfected with Sja-miR-71a and NC mimics, respectively and cultured for 24 h. The 1.5 × 10^6^ cells transfected with Sja-miR-71a were inoculated to the right scapula of mice, while the cells with NC mimics to the left scapula, respectively. Tumor volume was measured at days 2, 4, 6 and 8, respectively. The mice were sacrificed at day 8 for measurement of tumor weight and tumor volume using the formula: 1/2 × L × S^2^, where L is the longest diameter of tumor and S is the shortest diameter of tumor. The abundance of Sja-miR-71a was detected by qPCR, and expression of Ki67 and CD34 were examined by Immunohistochemistry (IHC) as described previously (Wang et al., 2013). The antibody against Ki67 and CD34 was used (1:50 dilution).

### Statistical Analysis

The data that are shown are the mean ± SD of at least three independent experiments. The differences were considered statistically significant at p <0.05 using one-way ANOVA, analyzed by GraphPad Prism 9.0 software.

## Results

### Identification of sja-miRNAs That Suppressed Tumor Cell Growth

We previously performed screening of a batch of schistosome miRNAs and have identified several *Sja-miRNAs* with antitumor activity such as Sja-miR-3096 (Lin et al., 2019) and Sja-miR-7-5p (Hu et al., 2019). Here, we performed further screening of additional 57 synthetic *Sja-miRNAs* mimics using human hepatoma cell line HepG2. Each sja-miRNA mimic was transfected into the tumor cell and cell cycles of the transfected cells were evaluated by FACS at 48 h post-transfection. We found four of the Sja-miRNAs including Sja-miR-71a, Sja-miR-3005, Sja-miR-3006, and Sja-miR-3044 that displayed significant arrest of cell cycle at G0/G1 phase (**Figure S1**). We selected Sja-miR-71a for further investigation as it is highly expressed at a variety of developmental stages of the parasite (Xue et al., 2008) and also present in the EVs of the *S. japonicum* eggs (Zhu et al., 2016), and showed the strongest inhibitory effect on cell cycle of the tumor cell among the four sja-miRNAs as well.

### Inhibitory Effect of Sja-miR-71a on Tumor Cell Proliferation *In Vitro*

**Figure 1:**
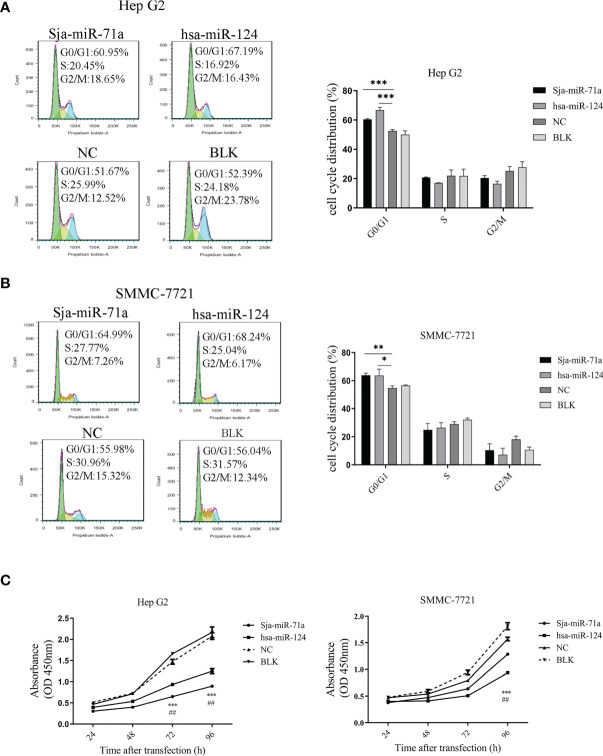
Inhibitory effect of sja-miR-71a on tumor cell *in vitro*. The human HepG2 and SMMC-7721 tumor cell lines were transfected with sja-miR-71a, hsa-miR-124 or NC mimics, respectively, and collected at 48 h post-transfection for analyses of cell cycle by FACS in HepG2 **(A)** and SMMC-7721 **(B)**, and proliferation by CCK8 assays **(C)**. The hsa-miR-124 was used as positive control. *p <0.05, **p <0.01, ***p <0.001, ^##^p < 0.01, positive control compared to NC.

To further determinate the effect of Sja-miR-71a on hepatoma cell *in vitro*, we chose two human hepatoma cell lines, HepG2 and SMMC-7721 (Chang et al., 2010), and used hsa-miR-124 as a positive control because of its antitumor activity (Silber et al., 2013; Cai et al., 2018; Xu et al., 2020). FACS analyses showed that transfection of Sja-miR-71a mimics significantly arrested cell cycle at G0/G1 phase of both tumor cell lines compared with the NC or blk (transfection reagents only) cohorts (**Figures 1A, B**). CCK-8 assays showed that Sja-miR-71a obviously inhibited cell proliferation of both HepG2 and SMMC-7721 in a time-dependent manner (**Figure 1C**). A similar inhibitory effect on the tumor cell growth was observed in a murine tumor cell line of hepa1-6 transfected with Sja-miR-71a mimics (**Figure S2A**). These data indicated that the Sja-miR-71a suppressed the growth of various tumor cell lines such as HepG2, SMMC-7721, and hepa1-6.

### Sja-miR-71a-Mediated Inhibition of Tumor Cell Migration

**Figure 2:**
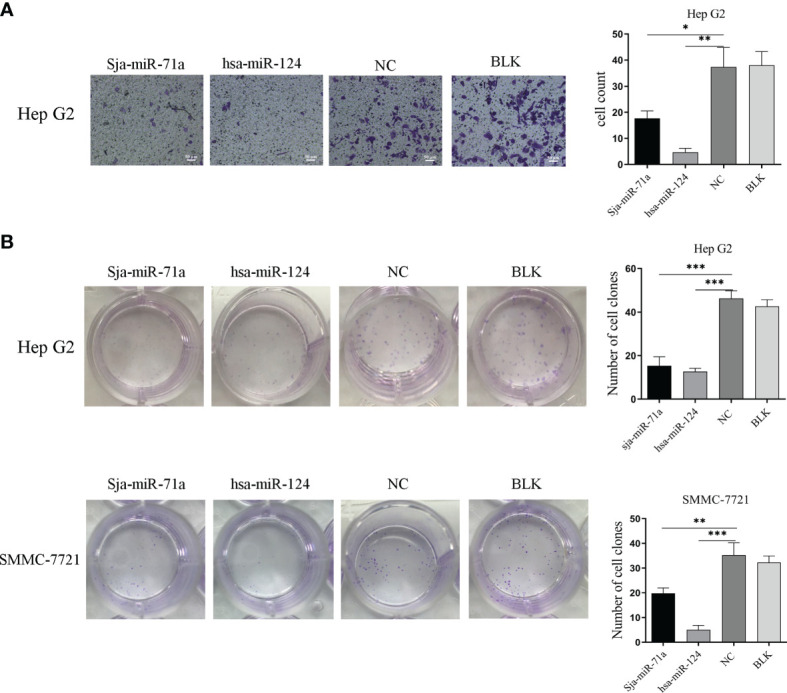
Sja-miR-71a-mediated inhibition of tumor cell migration and colony formation. **(A)** The HepG2 cell transfected with sja-miR-71a, hsa-miR-124 or NC mimics, respectively, was evaluated by Transwell assays using the Transwell inserts without Matrigel coating and cell counts were presented on the histogram. **(B)** Both transfected HepG2 and SMMC-7721 were evaluated for colony formation by Colony formation assay. *p <0.05, **p <0.01, ***p <0.001, compared to NC group.

To investigate if Sja-miR-71a exert inhibitory effect on cell migration, we performed Transwell assays using the Transwell inserts without Matrigel coating and showed that HepG2 transfected with Sja-miR-71a mimic displayed significant reduction of migration to the low chamber compared with the NC or blk cohorts (**Figure 2A**), but Sja-miR-71a has no effect on migration of SMMC-7721 cell and also on the hepa1-6 (**Figure S2B**). We further evaluated effect of Sja-miR-71a on colony formation of both HepG2 or SMMC-7721 cells. The results showed that HepG2 transfected with Sja-miR-71a mimics displayed significant decrease in the colony formation compared with the controls (**Figure 2B**) and similar outcome was observed in both SMMC-7721 (**Figure 2B**) and hepa1-6 cell (**Figure S2C**). These results indicated that Sja-miR-71a can suppress tumor cell migration and colony formation.

### Sja-miR-71a is Present in the Infected Hepatocytes

**Figure 3:**
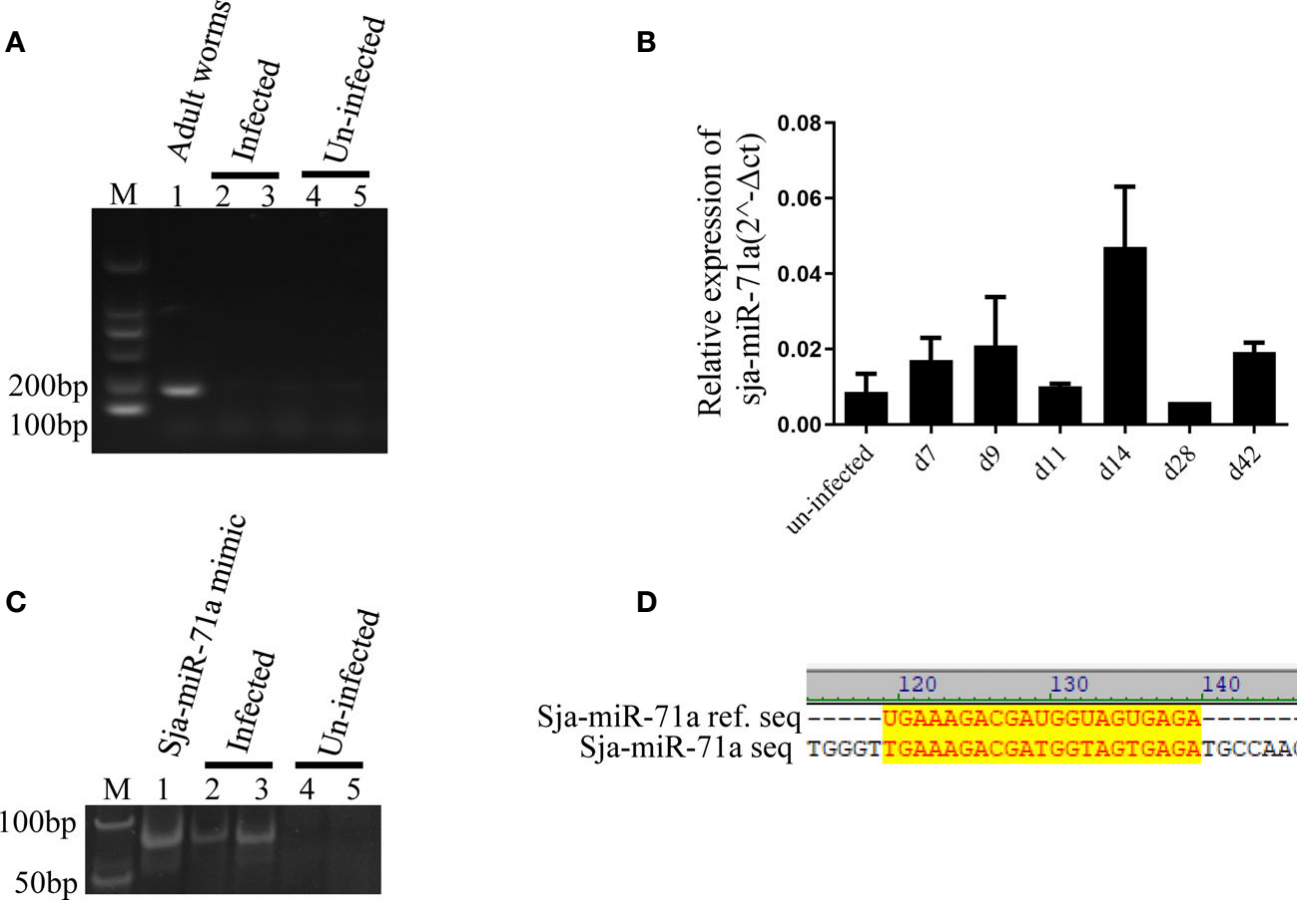
Presence of Sja-miR-71a in the infected hepatocytes. **(A)** PCR analyses of parasite NADH gene excludes contamination with parasite RNA. Lane 1: sample of parasite positive control; Lanes 2 and 3: samples of hepatocyte of mice infected with *S. japonicum* (infected); Lanes 4 and 5: samples of hepatocytes of normal mice (uninfected); **(B)** qPCR analyses of sja-miR-71a in the hepatocytes of mice during the parasite infection. **(C)** The PAGE electrophoresis of PCR products from the samples of *S. japonicum* infected (Lanes 2 and 3) or normal mice (Lanes 4 and 5) hepatocytes. Sja-miR-71a mimic were used as positive control (Lane 1). **(D)** The sequence alignment of reference sja-miR-71a and PCR products of infected hepatocytes.

We next investigated if the Sja-miR-71a is present in the host hepatocytes during *S. japonicum* infection, we separated hepatocytes from liver of infected mice as previously described (He et al., 2015). Total RNA of the hepatocytes was extracted for PCR or qPCR analyses. We excluded potential contamination with the parasite RNA by PCR detection of the parasite gene (NADH) in the samples and was not detected (**Figure 3A**). We then performed detection of Sja-miR-71a in the hepatocyte samples at different time-points by qPCR, and showed that Sja-miR-71a was detectable at days 7, 9, 14, and 42 post infection (**Figure 3B**). The presence of the Sja-miR-71a was also validated by PAGE electrophoresis of PCR products showing the product bands in the infected sample but not in the control sample (**Figure 3C**). Furthermore, sequencing of the PCR products showed identical to the sequence of Sja-miR-71a (**Figure 3D**).

### Identification of Target Genes of Sja-miR-71a

**Figure 4:**
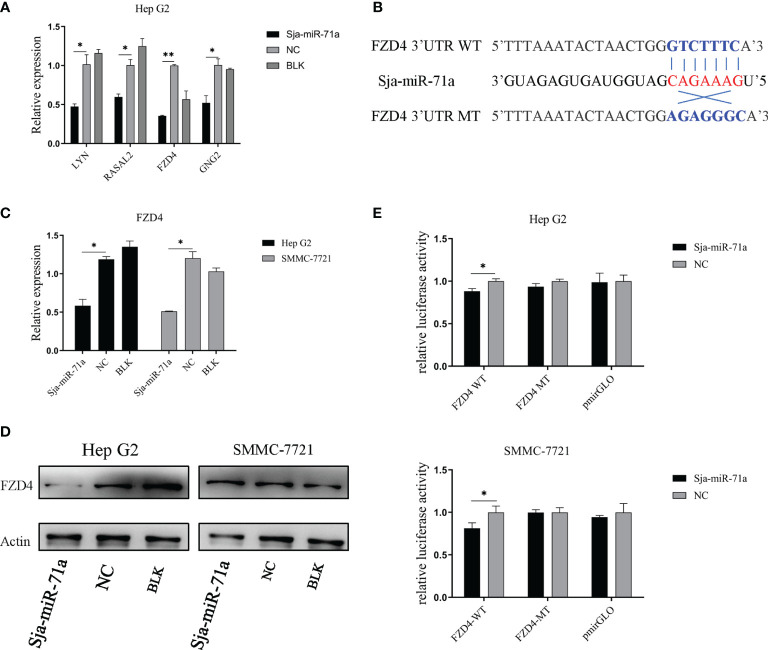
FZD4 is a target gene of sja-miR-71a. **(A)** qPCR validation of 4 target gene candidates in the HepG2 human tumor cell line transfected with sja-miR-71a or NC mimics at 48 h post-transfection. **(B)** The schematic diagram of binding site sequence between wild-type or its mutant of *FZD4* 3’UTR and seed region of sja-miR-71a. **(C)** The down-regulation of FZD4 expression in both HepG2 and SMMC-7721 cell lines transfected sja-miR-71a at mRNA level **(C)** and at protein level in HepG2 **(D)**. **(E)** Dual-luciferase reporter assays was performed to measure activity of the luciferase reporter gene. *p <0.05, **p <0.01, compared to NC group.

To identify the target genes of Sja-miR-71a, we used two publicly available websites miRDB (Wong and Wang, 2015) and RNAhybrid (Kruger and Rehmsmeier, 2006) to predict the potential target genes of Sja-miR-71a. A total of 413 human target genes were predicted (**Table S3**). We further used DAVID (Huang Da et al., 2009b; Huang Da et al., 2009a) to search for those genes from the predicted target genes that are related to tumor, and found the target genes of *LYN*, *RASAL2*, *FZD4*, and *GNG2* for further study (Choi et al., 2010; Yajima et al., 2012; Ma et al., 2017; Pan et al., 2018). We detected expression level of the four genes in HepG2 transfected with Sja-miR-71a or NC mimics at 48 h post-transfection. As shown in **Figure 4A**, comparing with NC or blk group, expressions of the four genes were significantly reduced. As *FZD4* is a key receptor of Wnt signaling pathway and its potential function in carcinoma, we then focused on this gene for further investigation. Sequence analyses showed a binding site at the 3ʹ-UTR of *FZD4* (**Figure 4B**). The qPCR analyses confirmed that transfection of both HepG2 and SMMC-7721 with Sja-miR-71a dramatically decreased mRNA expression level of *FZD4* (**Figure 4C**), while Western blot analyses showed significant reduction at protein level of the FZD4 in HepG2 (**Figure 4D**). To verify the *FZD4* gene as the target of Sja-miR-71a, we constructed several plasmids that expressing the luciferase reporter, in which the firefly luciferase gene is fused to the 3ʹ-UTR of *FZD4* gene (FZD4-WT), and also the mutants (FZD4-MT) where the binding site were mutated. Both HepG2 and SMMC-7721 cells were simultaneously transfected with the relevant plasmids and Sja-miR-71a mimics or NC mimics. As shown in **Figure 4E**, a significant reduction of the luciferase activity was detected in both of cells transfected with the FZD4-WT but not with the FZD4-MT.

**Figure 5:**
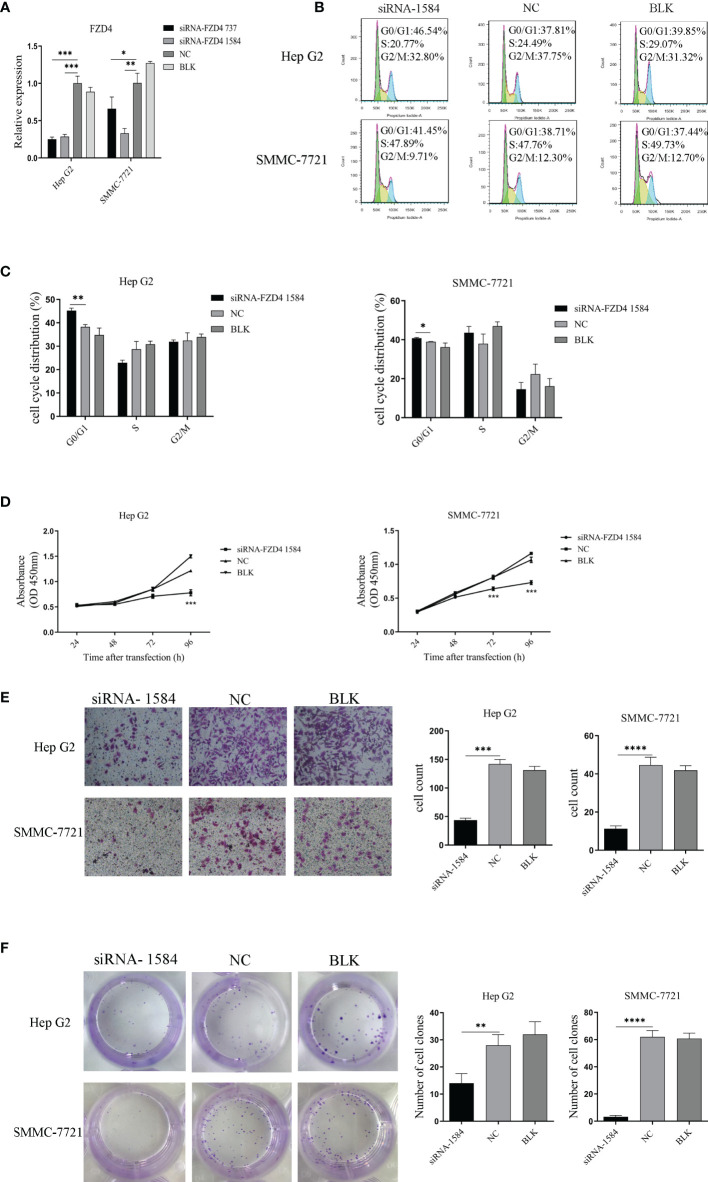
SiRNA-mediated knockdown of *FZD4* expression inhibits hepatoma cell proliferation and migration. **(A)** Both HepG2 and SMMC-7721 cells were transfected with siRNA-FZD4 737 and siRNA-FZD4 1584, respectively. The mRNA level of *FZD4* gene was detected by qPCR. **(B)** The hepatoma cells were transfected with siRNA-FZD4 1584 and analyzed for the cell cycle **(B, C)** and also for cell proliferation by cck8 assays **(D)**. Transfection of both HepG2 and SMMC-7721 cells with siRNA-FZD4 1584 were performed for analyses of the cell migration **(E)** and colony formation **(F)**. The cell count and the number of clones were shown in histogram, respectively. *p <0.05, **p <0.01, ***p <0.001, ****p < 0.0001.

To determine if Sja-miR-71a-mediated antitumor effect is through *FZD4*, we designed *FZD4* small interfering RNAs (siRNAs), named siRNA-737 and siRNA-1584. The qPCR detection indicated both siRNA-737 and siRNA-1584 significantly reduced *FZD4* expression in the tumor cell lines (**Figure 5A**). As the siRNA-1584 revealed high inhibition, we chose this siRNA for further studies. As expected, transfection of both HepG2 and SMMC-7721 cells with the siRNA-1584 led to cell cycle arrest at G0/G1 phase (**Figures 5B, C**). The cck-8 assays revealed the siRNA-1584 significantly inhibited the tumor cell proliferation compared to the siRNA-NC or blk (**Figure 5D**). In addition, Transwell (**Figure 5E**) and Colony formation assays (**Figure 5F**) demonstrated transfection with the siRNA-1584 significantly blocked cell migration and colony formation of both HepG2 and SMMC-7721. The phenotypes of the cells treated with FZD4 siRNAs were similar to those of Sja-miR-71a mimics-treated cells, suggesting that the FZD4 be the target gene of Sja-miR-71a and mediated anti-tumor effects.

### Sja-miR-71a Suppressed Tumor Growth *In Vivo*

**Figure 6:**
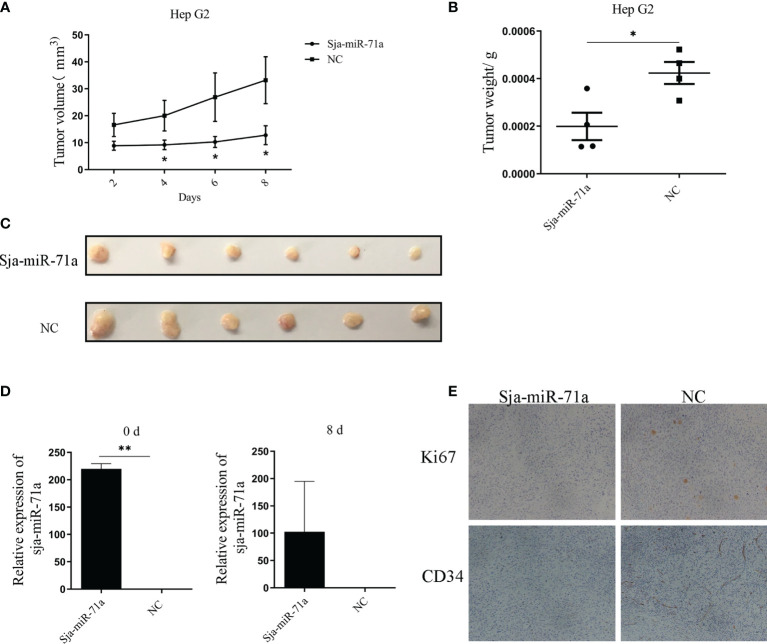
Sja-miR-71a suppressed tumor growth *in vivo*. The HepG2 cells were transfected with Sja-miR-71a mimics *in vitro*. The cells were collected after 24 h cultivation, and subcutaneously inoculated to nude BALB/c nice. The tumor growth curve **(A)**, and tumor weight **(B)** and volume **(C)** on day 8 post-inoculation were presented respectively. Besides, abundance of Sja-miR-71a in tumor at day 0 and 8 post-inoculation was detected **(D)**. Both Ki67 and CD34 expression in tumor tissue were detected by IHC **(E)**. *p <0.05, **p <0.01.

To further explore if the antitumor effect of Sja-miR-71a on the tumor cells *in vivo*, the HepG2 cells were transfected with Sja-miR-71a or NC mimics and then inoculated subcutaneously into the right and the left scapula of BALB/c nude mice, respectively. The results showed growth of the tumor cells transfected with the Sja-miR-71a mimics was obviously slower than that with NC mimics (**Figure 6A**). In addition, both tumor weight and volume were decreased in mice inoculated with tumor cells transfected with Sja-miR-71a compared with the control mice (**Figures 6B, C**). As shown in **Figure 6D**, the Sja-miR-71a was detectable in the tumors of mice transfected with this miRNA at day 8 after injection. Meanwhile, we detected the expression levels of both Ki67 and CD34 using immunohistochemistry (IHC) and showed that the protein level of Ki67 and CD34 were significantly decreased in tumor cells transfected with Sja-miR-71a compared with that in the NC control (**Figure 6E**). These data indicated that Sja-miR-71a suppressed hepatoma cell growth *in vivo*.

## Discussion

Human schistosomiasis is caused by several species of schistosome worms, including *S. mansoni*, *S. japonicum* and *S. haematobium* (Colley et al., 2014). Although infection with *S. haematobium* is associated with bladder cancer (Hashem et al., 1961; Mostafa et al., 1999; Zaghloul, 2012), it is less evident and controversial that infection with *S. japonicum* is associated with hepatocellular carcinoma (HCC). The case–control studies conducted in an endemic area of schistosomiasis japonica suggested that infection with *S. japonicum* had no directive relationship with HCC (Tokoro and Koganezawa, 1976; Inaba et al., 1984; Guo and Lu, 1987). Chronic infection of *S. japonicum* can lead to liver fibrosis, and eventually to liver cirrhosis, which could be high risk factors for HCC, but it does not seem to be the case in *S. japonicum* schistosomiasis. Thus, we speculated that some factors such as miRNAs and proteins derived from *S. japonicum* infection could be beneficial to host to strengthen resistance to certain host disease such as cancer.

Accumulated evidence displayed that miRNAs as oncogenes were involved in occurrence and development of various cancers (Hayes et al., 2014; Cao et al., 2018; Valencia et al., 2020). However, there were also many miRNAs identified in the past decade that exerted anti-tumor activity and revealed therapeutic effect on a variety of tumors (Xu et al., 2012; Jiang et al., 2016; Ishii et al., 2018). Moreover, recent studies suggested exogenous miRNAs derived from plants and parasites can be delivered to recipient cells and regulated tumor-related genes to exert antitumor effects. In the present study, we demonstrated that a specific *S. japonicum* miRNA, Sja-miR-71a, which exists in host liver cells during infection, has anti-tumor effect on hepatoma cells through suppression of growth and migration of tumor cell by cross-species regulation of *FZD4* gene. The Sja-miR-71a arrested cell cycle of both HepG2 and SMMC-7721 tumor cell lines at G0/G1 phase. Importantly, Sja-miR-71a-mediated suppression of the tumor growth was also observed in the xenograft nude mouse model. Further investigation of the mechanisms underlying this antitumor activity revealed that Sja-miR-71a exerts its function by targeting *FZD4* gene that is a receptor of Wnt pathway and associated with cancer cell viability and migration (Gupta et al., 2010). Therefore, our data suggested Sja-miR-71a is a tumor suppressor miRNA that might acts as a potential therapeutic target for cancer.

Based on bioinformatics analysis, Sja-miR-71a ortholog is also present in other schistosome species such as *S. mansoni*. In this study, we consider Sja-miR-71a as a schistosome-specific miRNA because there is no Sja-miR-71a ortholog in the hosts such as human and mouse. In addition, we mainly focused, in the present study, on the role of Sja-miR-71a in the regulation of host cellular functions rather than that in the parasite. Thus, further study should be considered to demonstrate its target genes in the parasite and illustrate potential role of Sja-miR-71a in the worm development and parasitism.

The human Frizzled-4 (*FZD4*) is the member of FZD family which encodes a 7-comparment trans-membrane type receptor *FZD4* (Gupta et al., 2010). The aberrantly upregulated expression of the gene was observed in various human cancers, such as prostate cancer and bladder cancer (Ueno et al., 2012; Formosa et al., 2014).The upregulation of *FZD4* expression was associated with tumor proliferation, migration and invasion, which was mediated by Wnt/β-catenin pathway (Yang et al., 2018; Chen et al., 2019). However, *FZD4* expression in HCC has not been characterized yet. In this study, we demonstrated that downregulation of *FZD4* in hepatoma cell by the *FZD4* siRNA inhibited the cell growth and migration, similar to the Sja-miR-71a-mediated antitumor activity. These data suggested that Sja-miR-71a exerted anti-tumor effects on hepatoma cells through downregulation of *FZD4* gene expression. It is interesting to note that Sja-miR-71a-mediated inhibitions of the migration of HepG2, SMMC-7721 and hepa1–6 generated different phenotypes. The mechanism behind this phenomenon is not clear. We speculated that it might be related to the migration ability and the endogenous expression levels of the target gene of those cell lines. In addition, MIR-71 has been identified in *Ascaris*, *F. hepatica*, *Opisthorchis* and many other parasites, but was lost in gnathostomes. The natural role of Sja-miR-71a in the biology of parasite and its host interaction has not been elucidated yet. It should be noted that the data on the Sja-miR-71a-mediated antitumor activity in this study was obtained based on transfection of Sja-miR-71a mimics to the cells *in vitro*, which should led to much higher levels of the transfected Sja-miR-71a than that of the endogenously expressed miRNA in the cells. Further studies would be considered to use alternative approach to tightly regulate expression of this miRNA within cell using regulatory vector to determine various levels of Sja-miR-71a on its antitumor activity.

In summary, we identified a schistosome miRNA, the Sja-miR-71a, that is present in host hepatocytes during the parasite infection. The schistosome-derived miRNA exerts an antitumor effect on hepatoma cell through arresting cell cycle at G0/G1 phase and suppression of tumor cell migration. The Sja-miR-71a-mediated suppression of tumor growth *in vivo* suggested that application of these exogenous miRNAs potentially provide a novel approach for cancer therapy.

## Data Availability Statement

The original contributions presented in the study are included in the article/**Supplementary Material**. Further inquiries can be directed to the corresponding author.

## Ethics Statement

The animal study was reviewed and approved by the Guide for the Care and Use of Laboratory Animals of the National Institutes of Health, the Internal Review Board of Tongji University School of Medicine.

## Author Contributions

PJ and WP conceived and designed the study. PJ, JW, and SZ performed the experiments. CH, YL, and WP analyzed the data. PJ and WP wrote the manuscript. All authors listed have made a substantial, direct, and intellectual contribution to the work and approved it for publication.

## Funding

This study was supported by the National Natural Science Foundation of China (81972985).

## Conflict of Interest

The authors declare that the research was conducted in the absence of any commercial or financial relationships that could be construed as a potential conflict of interest.

## Publisher’s Note

All claims expressed in this article are solely those of the authors and do not necessarily represent those of their affiliated organizations, or those of the publisher, the editors and the reviewers. Any product that may be evaluated in this article, or claim that may be made by its manufacturer, is not guaranteed or endorsed by the publisher.
