# Supplementary material for: Identification of a Schistosoma japonicum MicroRNA That Suppresses Hepatoma Cell Growth and Migration by Targeting Host FZD4 Gene
Source: Front Cell Infect Microbiol. 2022 Jan 31;12:786543. doi: 10.3389/fcimb.2022.786543 (PMC8842725; doi:10.3389/fcimb.2022.786543)
Supplement: Supplementary file 1 [file Table_1.doc]

**Supplementary table**

| **Table S1 Sequence of miRNAs and siRNAs** | | | |
| --- | --- | --- | --- |
| **name** | **sequence** | **name** | **sequence** |
| sja-miR-3010 | ACACGAACGGGAUAGCUGCU | sja-miR-3041 | CCACGGACUAUUAGAUGGGC |
| sja-miR-3078 | UCCGUGCUGAGAUUUCGUCAA | sja-miR-3044 | CGCGUGGAUCUGUCACACAUUA |
| sja-miR-3082 | UCUACCAAUGUGGCUCAGACU | sja-miR-3045 | CGUGGACAGCUACGUGAGUUU |
| sja-miR-3083 | UCUCCAACGAUGACUGAUGGU | sja-miR-3046 | CUAUUGAUUUGUUAUGGACUGA |
| sja-miR-3088 | UGACUGCAGAGCCCAAGGGA | sja-miR-3047 | CUCCCCAGAUACGUGAAGCGU |
| sja-miR-3103 | UGGCGCUUAGUAGAAUGUCACCG | sja-miR-3049 | CUGGCAGGUAGGUUUCCCGA |
| sja-miR-3007 | AAGUCGUACUGCUGACUGCAU | sja-miR-3054 | GGAAGGGCGAGAGCAGACAGUUUU |
| sja-miR-3015 | AGAAGACUGCCGCUCGUUUUA | sja-miR-3055 | GGAGGAACCUUGGACGAUAAA |
| sja-miR-3026 | AGGGCGAAGUCGUACUGCUGU | sja-miR-3056 | GGGGGUUGUUGUGGAGAUAGUUU |
| sja-miR-3037 | AUGUUCGGGAUGCGGUUUUCU | sja-miR-3068 | UACGCAAUGAUCGUGAGCAGUU |
| sja-miR-3050 | GAUAGAAGAUUGCCGCUCGUUU | sja-miR-3085 | UCUGAGGGCGAAGUCGUACUU |
| sja-miR-3067 | UACCGACUGCCUCGACGAGCUU | sja-miR-3089 | UGAGCCGGUGGGCCCUGAGUUU |
| sja-miR-3130 | UAGAAGACUGCCGCUCGUUUA | sja-miR-3090 | UGAGUCAUGUUGGUAGAUGUU |
| sja-miR-1732 | GACCGUGUUGCUGCAGGAGC | sja-miR-3091 | UGAUGUCGUCUGACAGGCUGU |
| sja-miR-2f | UCACAGCCAAUAUUGAUACCCG | sja-miR-3093 | UGCUCUGUGUUGAAUUUGGGUAUC |
| sja-miR-3001-a | UCAAAAUCGAUUGCAAUGGCU | sja-miR-3097 | UGGACUAAGUCAUGUUGGUU |
| sja-miR-3001-b | UCAAAAUCGUUGGCAAUGGCA | sja-miR-3102 | UGGCAGAUUGUCUUCGGAAUUU |
| sja-miR-3004 | AACAGAGACUGAUCAAUUCC | sja-miR-3104 | UGGGCAUAGGGCUAGCAUCCU |
| sja-miR-3005 | AAGGAACAACUGCUUGAAGC | sja-miR-3110 | UGUAGGCUGCGUGGUUGGGGUUU |
| sja-miR-3006 | AAGGUGUUUCCCUCGGACAAA | sja-miR-3113 | UGUUCCAGGACCUGUCGCAGUUU |
| sja-miR-3008 | AAUGCGCACUGCUGAAGAGUUU | sja-miR-3118 | UUGACACGAGGCGGACUGAG |
| sja-miR-3011 | ACGAAGACGACUGAGUUCGA | sja-miR-3119 | UUGAUGUUCACAGUGGGAUUUU |
| sja-miR-3013 | ACUGCAUAGGCGUAGCGGGU | sja-miR-3121 | UUGGGCUGAGCCAUGUUAAUU |
| sja-miR-3020 | AGAGGGUCCUGGGUUCGAUCUU | sja-miR-3122 | UUUAGUGCGAGCGCGUAUUUUU |
| sja-miR-3025 | AGGGACAACUGCUUGAGGCCAA | sja-miR-3125 | UUUUCGGCCAUCGGUAUUUGU |
| sja-miR-3027 | AGGGCGGUUAUCUCUUCCCA | sja-miR-3126 | UUUUGUUAUGCAUACACGUGU |
| sja-miR-3029 | AGGGUGAGGUGUGACAUUUUUU | sja-miR-5 | UAAGGAAUUAGAGAUGUGUUU |
| sja-miR-3038 | CAAAGGACGAGUGUACCGUGU | sja-miR-71a | UGAAAGACGAUGGUAGUGAGAUG |
| sja-miR-3040 | CAAGUGACGCAAACUGGAAA | hsa-miR-124 | UAAGGCACGCGGUGAAUGCCAA |
|  |  |  |  |
| si-FZD4 737 sense | AGUCUGAACUGCAGCAAAUTT | si-FZD4 1584 sense | GGACAAAGACAGACAAGUUTT |
| si-FZD4 737 antisense | AUUUGCUGCAGUUCAGACUTT | si-FZD4 1584 antisense | AACUUGUCUGUCUUUGUCCTT |
| NC sense | UUCUCCGAACGUGUCACGUTT |  |  |
| NC antisense | ACGUGACACGUUCGGAGAATT |  |  |

| **Table S2 Sequences of primers used for RT-qPCR and PCR** | | |
| --- | --- | --- |
| Gene | Name | Sequence (5'-3') |
| Sja-miR-71a | RT-stem-loop primer | CTCAACTGGTGTCGTGGAGTCGGCAATTCAGTTGAGCATCTCA |
|  | Forward | ACACTCCAGCTGGGTTGAAAGACGATGG |
|  | Reverse | CTGGTGTCGAGTCGGCAA |
| hsa-u6 | RT-stem-loop primer | CGCTTCACGAATTTGCGTGTCA |
|  | Forward | GCTTCGGCAGCACATATACTAAAAT |
|  | Reverse | CGCTTCACGAATTTGCGTGTCAT |
| hsa-GAPDH | Forward | CAGGGCTGCTTTTAACTCTGGTAA |
|  | Reverse | GGGTGGAATCATATTGGAACATGT |
| hsa-FZD4 | Forward | GTGTCACTCTGTGGGAACCAA |
|  | Reverse | GGCTGTATAAGCCAGCATCAT |
| hsa-LYN | Forward | GCTTTTGGCACCAGGAAATAGC |
|  | Reverse | TCATGTCGCTGATACAGGGAA |
| hsa-RASAL2 | Forward | AGCAGAAAGGTCCCCTCGTAG |
|  | Reverse | AGGGTGAGGTATTTGCAGTGT |
| hsa-GNG2 | Forward | AACACCGCCAGCATAGCAC |
|  | Reverse | CCTGTCGATATTGGCTTCCATCT |
| Sja-NADH | Forward | CGAGGACCTAACAGCAGAGG |
|  | Reverse | TCCGAACGAACTTTGAATCC |
| WT-FZD4 3’UTR | F1 | TCTAGTTGTTTAAACGAGCTCCAGGAAGTCCAGGGCGATAC |
|  | R1 | CCTGCAGGTCGACTCTAGACAGACAGCGCACCACAGAAGATG |
| MT-FZD4 3’UTR | F2 | ATACTAACTGGAGAGGGCAGATAGCAAAGCAATC |
|  | R2 | TCTCCCGCCAGTTAGTATTTAAAATGGTAAAG |
| Note: red letters mean mutation site. | | |

**Supplementary figures**

**
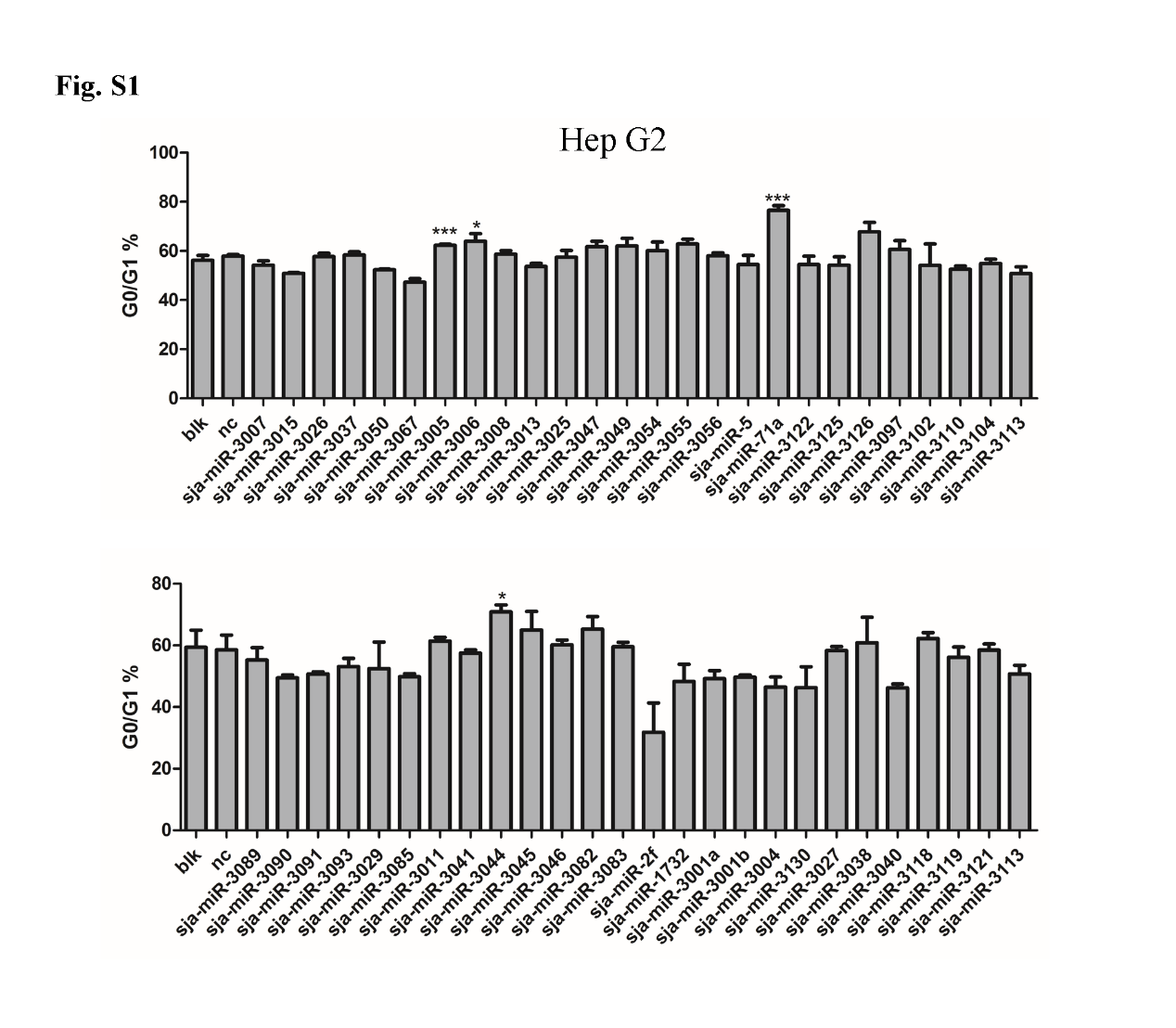
**

**Figure S1. Screening and identification of *S. japonicum* miRNAs that suppressed hepatoma cell cycle.**

HepG2 cells were transfected with sja-miRNA or NC mimics. At 48h post-transfection, cells were collected and analyzed by Flow Cytometry. Cell cycles at G0/G1 phase were analyzed by FlowJo software. *p<0.05, **p<0.01, ***p<0.001

**
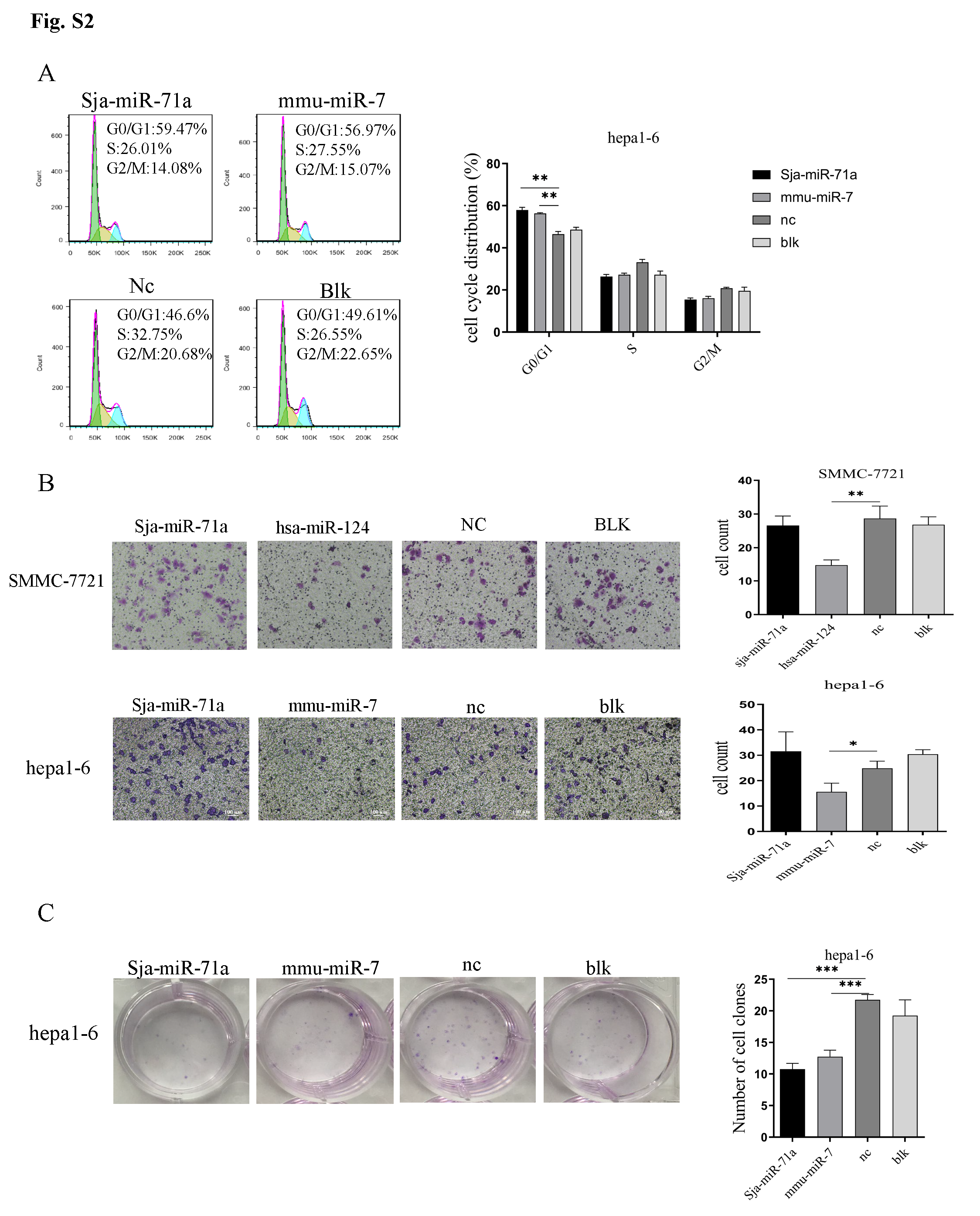
**

**Figure S2. The antitumor effect of sja-miR-7a on murine hepa1-6 and SMMC-7721.**

The murine hepatoma cell of hepa1-6 was transfected with sja-miR-71a, mmu-miR-7(as positive control) or NC mimics and analyzed for cell cycle at G0/G1 phase(A) and colony formation(C). However, sja-miR-71a had no inhibitory effect on migration of both SMMC-7721 and hepa1-6(B). *p<0.05, **p<0.01, ***p<0.001
